# Land-use intensification differentially affects bacterial, fungal and protist communities and decreases microbiome network complexity

**DOI:** 10.1186/s40793-021-00396-9

**Published:** 2022-01-06

**Authors:** Sana Romdhane, Aymé Spor, Samiran Banerjee, Marie-Christine Breuil, David Bru, Abad Chabbi, Sara Hallin, Marcel G. A. van der Heijden, Aurélien Saghai, Laurent Philippot

**Affiliations:** 1 Department of Agroecology, University Bourgogne Franche Comte, INRAE, AgroSup Dijon, Dijon, France; 2 Agroscope, Plant-Soil Interactions Group, Zurich, Switzerland; 3 Department of Biological Sciences, North Dakota State University, Fargo, 58102 USA; 4 ECOSYS, UMR INRAE, AgroParisTech, Thiverval-Grignon, France; 5 CNRS, Institute of Ecology and Environmental Sciences-Paris (iEES-Paris, UMR Sorbonne Université, CNRS, INRAE), Thiverval-Grignon, France; 6 Department of Forest Mycology and Plant Pathology, Swedish University of Agricultural Sciences, Uppsala, Sweden; 7 Department of Plant and Microbial Biology, University of Zurich, Zurich, Switzerland

**Keywords:** Land-use intensification, Microbial communities, Networks

## Abstract

**Background:**

Soil microbial communities are major drivers of cycling of soil nutrients that sustain plant growth and productivity. Yet, a holistic understanding of the impact of land-use intensification on the soil microbiome is still poorly understood. Here, we used a field experiment to investigate the long-term consequences of changes in land-use intensity based on cropping frequency (continuous cropping, alternating cropping with a temporary grassland, perennial grassland) on bacterial, protist and fungal communities as well as on their co-occurrence networks.

**Results:**

We showed that land use has a major impact on the structure and composition of bacterial, protist and fungal communities. Grassland and arable cropping differed markedly with many taxa differentiating between both land use types. The smallest differences in the microbiome were observed between temporary grassland and continuous cropping, which suggests lasting effects of the cropping system preceding the temporary grasslands. Land-use intensity also affected the bacterial co-occurrence networks with increased complexity in the perennial grassland comparing to the other land-use systems. Similarly, co-occurrence networks within microbial groups showed a higher connectivity in the perennial grasslands. Protists, particularly Rhizaria, dominated in soil microbial associations, as they showed a higher number of connections than bacteria and fungi in all land uses.

**Conclusions:**

Our findings provide evidence of legacy effects of prior land use on the composition of the soil microbiome. Whatever the land use, network analyses highlighted the importance of protists as a key element of the soil microbiome that should be considered in future work. Altogether, this work provides a holistic perspective of the differential responses of various microbial groups and of their associations to agricultural intensification.

**Supplementary Information:**

The online version contains supplementary material available at 10.1186/s40793-021-00396-9.

## Background

Agricultural intensification represents one of the major causes of biodiversity loss in the twenty-first century [1, 2]. Intensive land use also threatens ecosystem multifunctionality [3] and increases negative environmental impact, for example by increased greenhouse gas emissions [4] and nitrogen losses through leaching [5]. Land intensification is most often associated with shifts in vegetation, which in return can affect the soil microbiome. It is well known that above-ground vegetation influences below-ground microorganisms through, for example, the exudation of organic carbon or the modification of soil properties in close vicinity to plant roots [6–8]. Besides, intensive agricultural practices such as tillage, fertilization, and the use of pesticides can affect the soil microbiome [9–11]. Not only current but also past land-use can have effects that persist for decades, affecting the microbiome in contemporary land use [12, 13]. For example, a history of tillage or no tillage 60 years before conversion to longleaf pine savanna was found to differentially affect the diversity and composition of bacterial and fungal communities [13]. Since soil microbes are major players of the biogeochemical cycles and therefore tremendously important for ecosystem functions [14–16], it is important to understand how microbial communities are affected by increased land-use intensity, and to what extent the responses are modulated by legacy effects of prior land use.

Although the soil microbiome is highly diverse and complex, most studies investigating the impact of land-use intensification on microbial diversity have focused on either bacteria and/or fungi [17–20]. However, microbes are not a mere collection of independent populations, but form complex communities and the direct and indirect interactions between taxa appear to play a crucial role for the assembly of microbial communities, with consequences for ecosystem functioning [21–25]. For example, the often neglected protists are important in agricultural ecosystems as they control microbial populations through predation [26–28] and influence ecosystem functions *e.g.* nutrient cycling [29]. In particular, by grazing on bacteria, phagotrophic protists are increasing the mobilization of bacterial nitrogen and are therefore contributing to nitrogen mineralization [29, 30]. This exemplifies the close interaction across soil microbes from different domains and that such interactions can affect soil functioning. An integrated assessment of the soil microbiome is thus needed for a comprehensive understanding of the impact of land-use intensification on soil microorganisms. Co-occurrence networks are often used to decipher relationships between microorganisms [31]. Although co-occurrence patterns might not reflect the true complexity of microbial interactions, this integrative approach can help to better understand the consequences of changes in land use on microbial communities [31–33]. For example, fungal networks were more connected in organic farming than in conventional or no-till farming systems, which was concomitant with a decrease in root colonization by arbuscular mycorrhizal fungi [17]. However, studies investigating how land-use intensification modifies co-occurrence patterns within and especially across domains are still scarce.

Here, we investigated how land-use intensification and the legacy effects of prior land-use affect the diversity, structure and co-occurrence patterns within and between bacterial, protist and fungal communities. For this purpose, we used a long-term (> 12 years) replicated field experiment, where a gradient of land-use intensity was established, with high (continuous cropping with annual crops), medium (alternating annual crops with a temporary grassland) and low (perennial grassland) intensity. We hypothesized that (i) changes in land-use will lead to distinct responses across microbial groups with smallest differences between temporary and perennial grasslands if there are no legacy effects (i.e. no impact of the continuous cropping preceding the temporary grassland), and (ii) co-occurrence patterns of the soil microbiome will shift along the gradient of land-use intensity with a higher network complexity in the perennial systems since the annual rotation of short lived-seasonal crops is less likely to support the co-evolution of multitrophic interactions within the soil microbiota. Because soil microbes are key players in the cycling of nitrogen, which is the major nutrient limiting primary production in terrestrial ecosystems [34], we also investigated whether land-use intensification affected the abundances of functional microbial guilds involved in N-cycling.

## Methods

### Experimental design and soil sampling

Soil samples were collected from a long-term field experiment SOERE-ACBB (Systems of Observation and Experimentation in Environmental Research in Agro-ecosystems, Biochemical cycles and Biodiversity) at the INRAE experimental station located at Lusignan, France (46°25′12.91″ N; 0°07′29.35″ E). The soil is a Cambisol with loamy texture (105 g kg^−1^ sand, 727 g kg^−1^ silt and 168 g kg^−1^ clay). Organic carbon content and nitrogen levels are shown in Additional file 1 (table S1). The experiment was established in 2005 in a randomized complete block design divided in four blocks containing plots with a surface of 4000 m^2^ each (Additional file 1: Figure S1). Each block comprises the following treatments: a three-year crop rotation of maize-wheat-barley (CC), a three-year temporary grassland alternated with the three-year crop rotation (TG) and a permanent grassland (PG). In the crop rotation, conventional tillage (mouldboard ploughing) and fertilizer management was applied, whereas in the grasslands, the timing and rate of nitrogen application was guided by a nitrogen nutrition index between 0.9 and 1.0 to provide the lowest nitrogen amount for potential plant production [35, 36]. The dominant vegetation of the temporary and permanent grassland was composed of *Lolium perenne*, *Festuca arundinacea* and *Dactylis glomerata*. The land-use intensity was defined by cropping frequency (continuous cropping > alternating cropping with a temporary grassland > perennial grassland).

Sampling was carried out in November 2017, which was before sowing for CC and TG, and therefore after the last year of the temporary grassland for TG. Five soil cores were collected randomly from each replicated plot using a soil corer of 20 cm depth and 5 cm diameter to obtain 20 samples from each treatment (N total = 60 samples). Plant debris and roots were removed from soil samples, which were then homogenized and sieved at 4 mm. Soil relative moisture was determined by drying 10 g of fresh soil at 105 °C for 24 h.

***Assessment of microbial community composition and diversity***

DNA was extracted from the 60 soil samples using the DNeasy PowerSoil-htp 96 well DNA isolation kit (Qiagen, France). Analysis of the diversity and composition of the bacterial, protist and fungal communities were performed for all DNA extracts using Illumina sequencing of the 16S rRNA and 18S rRNA genes and the fungal ITS1 region. Amplicons were generated for all DNA extracts in two steps. In the first step, the V3-V4 hypervariable region of the bacterial 16S rRNA gene was amplified by polymerase chain reaction (PCR) using the fusion primers U341F (5’-CCTACGGGRSGCAGCAG-3’) and 805R (5’-GACTACCAGGGTATCTAAT-3’)[37]. The V4 hypervariable region of 18S rRNA gene was amplified by PCR using the primers EK-565F (5′-GCAGTTAAAAAGCTCGTAGT-3′) and 18S-EUK-1134-R–UnonMet (5′-TTTAAGTTTCAGCCTTGCG-5′) [38, 39]. Fungal ITS1 region was amplified using the primers ITS1F (CTTGGTCATTTAGAGGAAGTAA) and ITS2 (GCTGCGTTCTTCATCGATGC) primers [40, 41]. Sequencing was performed on MiSeq (Illumina, 2 × 250 bp and 2 × 300 bp) using the MiSeq reagent kit v2 and v3 (500 and 600 cycles, respectively).

***Sequencing and bioinformatic analysis***

Sequence data from the 60 soil samples was analyzed using an in-house developed Python pipeline (https://forgemia.inra.fr/vasa/illuminametabarcoding). Briefly, 16S rRNA gene, 18S rRNA gene and ITS sequences were assembled using PEAR [42] with default settings. Further quality checks were conducted using the QIIME 1 pipeline [43] and short sequences were removed (< 400 bp for 16S, < 450 bp for 18S and < 300 bp for ITS). Reference based and de novo chimera detection, as well as OTU clustering were performed using VSEARCH [44] and the adequate reference databases (SILVA representative set of sequences for 16S and 18S rRNA, and UNITE’s reference dynamic dataset for ITS). The identity thresholds were set at 94% for 16S rRNA gene-based on replicate sequencing of a bacterial mock community that contains 40 different bacterial strains for which we have the full-length 16S rDNA sequences [45] and 97% for 18S rRNA gene and ITS. Representative sequences for each OTU were aligned using PyNAST [46] and MUSCLE for 16S rRNA and 18S rRNA, respectively. Phylogenetic trees were constructed using FastTree [47]. For 16S, taxonomy was assigned using UCLUST [48] and the SILVA reference database 132 [49]. For 18S, taxonomy was assigned using PR^2^ database 4.11.1 [50]. For ITS, the taxonomy assignment was performed using BLAST [51] and the UNITE reference database (v.7–08/2016 [52]). Raw sequences were deposited at the NCBI under the BioProjects PRJNA741976, PRJNA741982 and PRJNA742156.

Based on taxonomic assignments, we filtered out OTUs from the 18S rRNA gene sequences that were non-protist (*i.e.* OTUs belonging to Fungi, Streptophyta, Mollusca and Porifera) and OTUs from ITS sequences that were non-fungal (*i.e.* OTUs belonging to Cercozoa). In total, 2 319 835 bacteria sequences, 1 632 206 protist sequences and 3 244 474 fungal sequences were obtained and assigned to 6 999, 3 867 and 1 866 OTUs, respectively. Bacterial, protist and fungal *α*-diversity metrics (*i.e*. observed species, Simpson’s reciprocal, Shannon as well as Faith’s Phylogenetic Diversity PD for bacteria and protist [53]) were calculated based on rarefied OTU tables (23,000 sequences per sample for 16S rRNA, 9000 sequences per sample for 18S rRNA and 35,000 sequences per sample for ITS). Bray–Curtis dissimilarity matrix were also computed to detect variations in the structure of microbial communities.

***Quantification of N-cycling bacterial and archaeal communities***

The abundances of total bacterial and fungal microbial communities as well as that of nitrogen cycle microbial guilds were estimated by real-time quantitative PCR (qPCR) assays. For each land use, the five DNA extracts from each replicated plot were pooled in equimolar amounts which corresponded to 4 composite samples per land use used as templates for the qPCR assays (n = 4). Total bacterial and fungal communities were quantified using 16S rRNA and ITS primers as described by Muyzer et al. (1993) [54] and White et al. (1990) [41], respectively. Marker genes for nitrification (*amoA* in archaea and bacteria) and denitrification (*nirK* and *nirS*) were quantified as described previously [55, 56]. Finally, *nifH* and *nrfA* genes were used to quantify communities involved in nitrogen-fixation [57] and dissimilatory nitrate reduction to ammonium (DNRA) [58, 59], respectively. The qPCR reactions were carried out using a ViiA7 (Life Technologies, USA) in 15 µl reactions containing 7.5 µL of Takyon Master Mix (Eurogentec, France), 1 µM of each primer, 250 ng of T4 gene 32 (QBiogene, France) and 1 ng of DNA. Two independent runs were performed for each real time PCR assay. Standard curves were obtained using serial dilutions of linearized plasmids containing appropriated cloned targeted genes from bacterial strains or environmental clones. PCR efficiency for the different assays ranged from 77 to 101%. No template controls gave null or negligible values. Inhibition in qPCR assays was tested by mixing soil DNA extracts with either control plasmid DNA (pGEM-T Easy Vector, Promega, France) or water. No inhibition was detected with the amount of DNA used.

***Statistical analyses***

Statistical analyses were conducted using R statistical software version 3.4.1 [60]. Differences in gene copy number (16S rRNA, ITS, bacterial and archaeal *amoA*, *nirK*, *nirS*, *nifH* and *nrfA*) were tested using the Kruskal–Wallis test followed by Dunn’s multiple comparison test (adjusted p-value < 0.05). Differences in the microbial *α*-diversity indices were tested using ANOVA followed by Tukey's honestly significant difference (HSD) test (p-value < 0.05) using the agricolae package [61]. Normality and homogeneity of the distribution of residuals were verified and log-transformations were performed when necessary. Permutational multivariate analysis of variance (PermANOVA) was carried out on the Bray–Curtis dissimilarity distance matrices using adonis function implemented in the vegan package [62]. Pairwise post hoc tests were conducted using the function pairwise.adonis from the pairwiseAdonis package [63] with Holm corrections. As sequencing data are usually sparse, low-abundance OTUs were filtered out by keeping OTUs that are present at a threshold of 0.04% in all samples. We also discarded the OTUs that were not found in at least 10 out of the 60 samples. This filtering step allows reducing the zero counts in sequencing datasets, which can inflate the number of false positive for the differential abundances analysis and spurious correlation between OTUs in network analysis. This resulted in 472 OTUs for 16S rRNA, 341 OTUs 18S rRNA and 218 for ITS. Differential abundance analysis of microbial community composition was conducted by pairwise comparisons between land uses of filtered count matrices (n = 20) using the DESeq2 package (FDR-corrected p-value < 0.00001, Additional file 2) [64]. Significantly discriminant OTUs were illustrated by ternary plots using ggtern package [65] and Upset plot using UpsetR package [66].

Networks were inferred using a sparse multivariate Poisson log-normal (PLN) model with a latent Gaussian layer and an observed Poisson layer using the PLNmodels package (Additional file 2) [67]. The best network was selected using a Stability Approach to Regularization Selection (StARS) [68], which performs a random subsampling of the input data to select a network with low variability in the selected edges. A specific normalization with the TSS (Total Sum Scaling) method was performed in order to take into account the heterogeneity of sequencing depth within and between microbial groups. All networks were constructed using filtered count matrices (*i.e.* 472 OTUs for 16S rRNA, 341 OTUs 18S rRNA and 218 for ITS). Bacterial, protist and fungal networks were inferred separately for each land use (n = 20) and for visualization purpose only partial correlations with |ρ|> 0.1 were considered. Inter-domain networks were inferred using all microbial groups for each land use (n = 20), and for visualization purpose partial correlations with |ρ|> 0.08 were visualized. Networks were then visualized using the Cytoscape software [69]. The NetworkAnalyser tool from Cytoscape was used to calculate network topological parameters (*i.e.* nodes, links, clustering coefficient and degree).

## *Results*

***Land-use intensification impacts soil microbial communities***

To characterize the soil bacterial, fungal and protist communities under continuous cropping (CC), alternating cropping with a temporary grassland (TG) and perennial grassland (PG), we used DNA metabarcoding targeting the bacterial, protist and fungal communities. Significant differences in *α*-diversity indices were observed for the bacterial community, with OTU richness, Shannon index, and phylogenetic diversity being higher in continuous cropping and temporary grassland than in the perennial grassland (Tukey’s test, p-value < 0.05, Additional file 1: Figure S2). By contrast, the *α*-diversity indices of the protist and fungal communities were similar across the three land-uses.

**Fig. 1:**
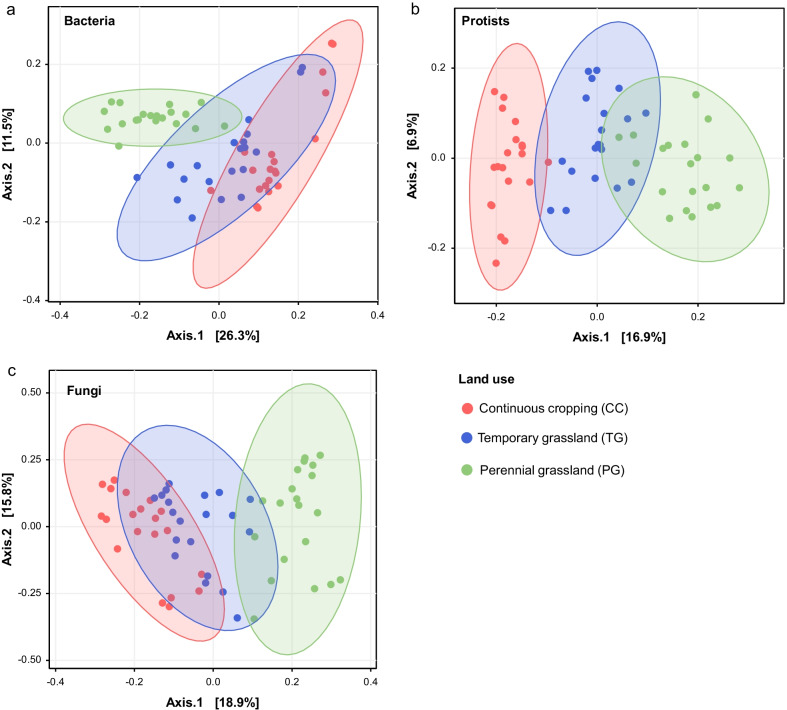
Principal Coordinates Analysis (PCoA) of the Bray–Curtis distance matrices of **a** bacterial 16S rRNA gene fragments, **b** protist 18S rRNA gene fragments and (**c**) fungal ITS1 amplicons showing shifts in community structure between continuous cropping (CC), temporary grassland (TG) and perennial grassland (PG) land-use

Comparison of *β*-diversity using Principal Coordinates Analysis (PCoA) of Bray–Curtis distances showed a clustering of samples according to management with 37%, 23% and 34% of the variance explained by the first two axes of the PCoA for bacterial, protist and fungal communities, respectively (Fig. 1). PermANOVA confirmed significant differences in the structure of the microbial communities between land uses (PermANOVA, p-value < 0.05). However, the structure of the bacterial and protist communities were more similar between continuous cropping and the temporary grassland than the perennial grassland (Additional file 1: Figure S3).

**Fig. 2:**
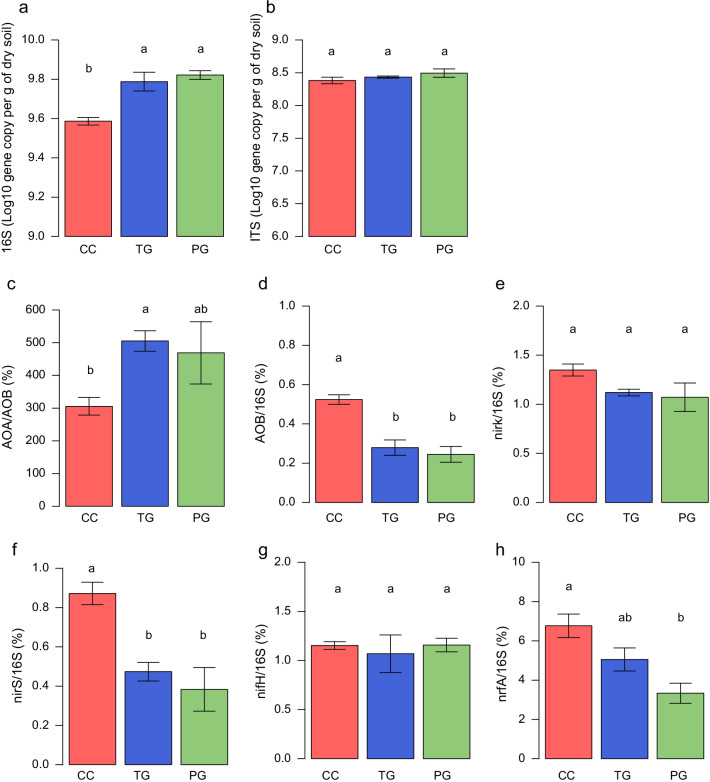
Abundances of **a** total bacteria (16S rRNA gene) and **b** fungal (ITS) in continuous cropping (CC), temporary grassland (TG) and perennial grassland (PG) soil samples (mean ± s.e.). The percentage of **c** ammonia-oxidizing archaea (AOA) to ammonia-oxidizing bacteria (AOB). Percentage of **d** AOB, **e** and **f** bacterial denitrifiers (*nirK* and *nirS*), **g** nitrogen-fixing communities (*nifH*) and **h** DNRA communities (*nrfA*) in the total bacterial community. Different letters above the bars indicate significant differences between land uses according to Dunn’s test (adjusted p-value < 0.05)

The abundance of the total bacterial community, determined by quantification of the 16S rRNA gene, was significantly lower under continuous cropping compared to temporary and perennial grasslands (Dunn’s test, adjusted p-value < 0.05, Fig. 2). For the N-cycling guilds, the percentage of DNRA bacteria (*nrfA)* within the total bacterial community (*nrfA*/16S rRNA gene abundance) increased along the land-use intensity gradient (Dunn’s test, adjusted p-value < 0.05, Fig. 2). The percentage of AOB and of *nirS*-denitrifiers were also higher in the continuous cropping, but significant differences were not observed for other N-cycling guilds (Dunn’s test, adjusted p-value < 0.05).

***Identifying differentially abundant OTUs***

**Fig. 3:**
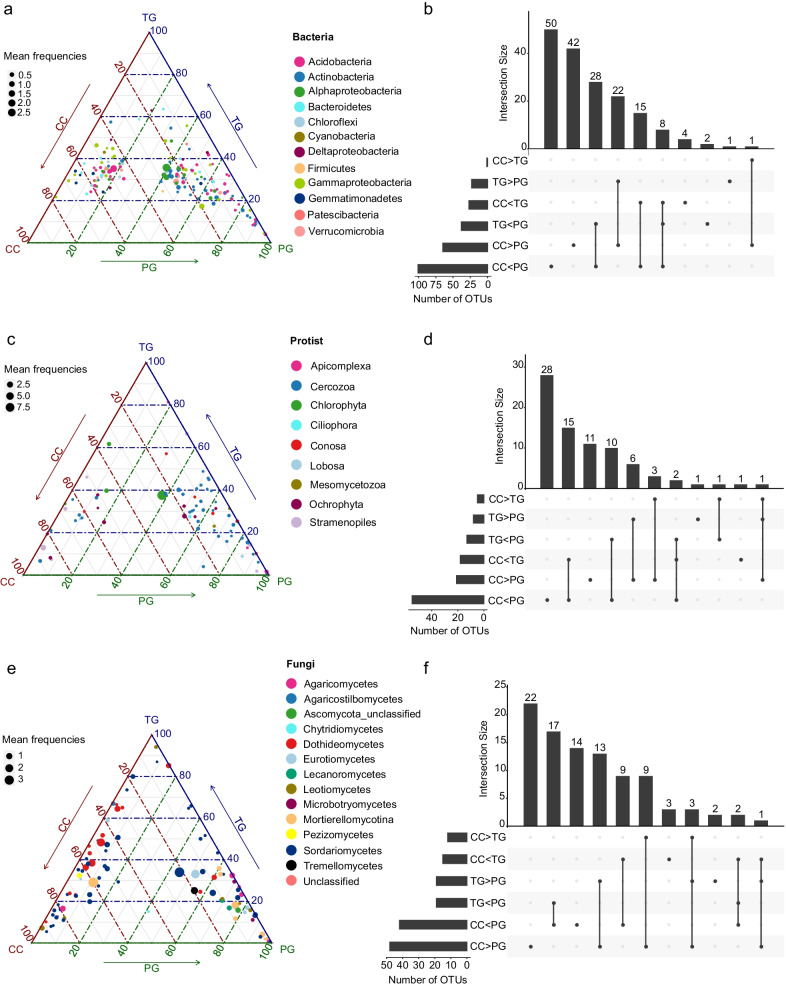
Ternary plots representing the composition of the microbial community under different land uses. Differential OTU abundances between land uses identified by DESeq2 are represented for **a** bacterial, **c** protist and **e** fungal communities. The position of each circle on the axis represents the contribution of the indicated land use to the relative abundance of each OTU. The size of the circle indicates the mean frequencies of each OTU in all samples. The colors indicate the affiliation of OTUs at the phylum or class levels. Upset plots showing the differentially abundant OTUs between land uses for **b** bacterial, **d** protist and **f** fungal communities. Each vertical bar shows the number of differentially abundant OTUs that are shared or unique between land use comparisons

Across all samples, the dominant bacterial phyla were Proteobacteria (33%), Acidobacteria (21%) and Actinobacteria (17%). Protists were dominated by Cercozoa (45%), Chlorophyta (15%) and Stramenopiles (14%), whereas Mortierellomycotina (42%) and Sordariomycetes (22%) represented the most abundant fungal taxa (Additional file 1: Figure S4). The ternary plots showed the distribution of the most abundant OTUs, with those present at similar abundances in all samples being near the center of the ternary plots (Additional file 1: Figure S5). Differential abundance analysis based on the most abundant OTUs (472 OTUs for 16S rRNA, 341 OTUs 18S rRNA and 218 for ITS), identified 173 bacterial OTUs with significant changes in abundances between land-uses (FDR-corrected P < 0.00001) (Fig. 3a). The strongest differences were observed between continuous cropping and permanent grasslands, with 166 discriminant bacterial OTUs and 101 of them being significantly more abundant under perennial grasslands than continuous cropping (Fig. 3b). By contrast, only 28 OTUs differed significantly between continuous cropping and temporary grasslands, with 27 OTUs increasing in the latter. OTUs belonging to Actinobacteria were the most impacted by land-use intensity, showing a decrease in relative abundances from 7 to 3% from the perennial grassland to the continuous cropping (Fig. 3a and Additional file 1: Figure S4 a). For the protist, 79 out of the 341 dominant OTUs differed significantly between the three land uses (Fig. 3c). The majority were differentially abundant between perennial grassland and continuous cropping (Fig. 3d), with a decrease in the relative abundances of 55 OTUs from 32 to 10%, respectively. The most impacted OTUs belonged to Apicomplexa and Cercozoa (Fig. 3 c and Additional file 1: Figure S4b). For the fungal community, 95 out of 218 fungal OTUs were significantly impacted by the land management (Fig. 3e). Similar to the patterns observed for bacteria and protist, the largest differences were between the perennial grassland and the continuous cropping with 42 OTUs being more abundant under perennial grassland (*e.g.* Agaricomycetes and Mortierellomycotina), and 48 OTUs mainly assigned to Dothideomycetes having higher abundances under continuous cropping (Fig. 3f and Additional file 1: Figure S4c). We found that only 4 OTUs belonging to fungi and protist were significantly increasing along the land-use intensity gradient, (CC > TG > PG), while 12 OTUs that belonged to all three domains were decreasing (CC < TG < PG).

***Co-occurrence networks within microbial groups are influenced by land use***

**Fig. 4:**
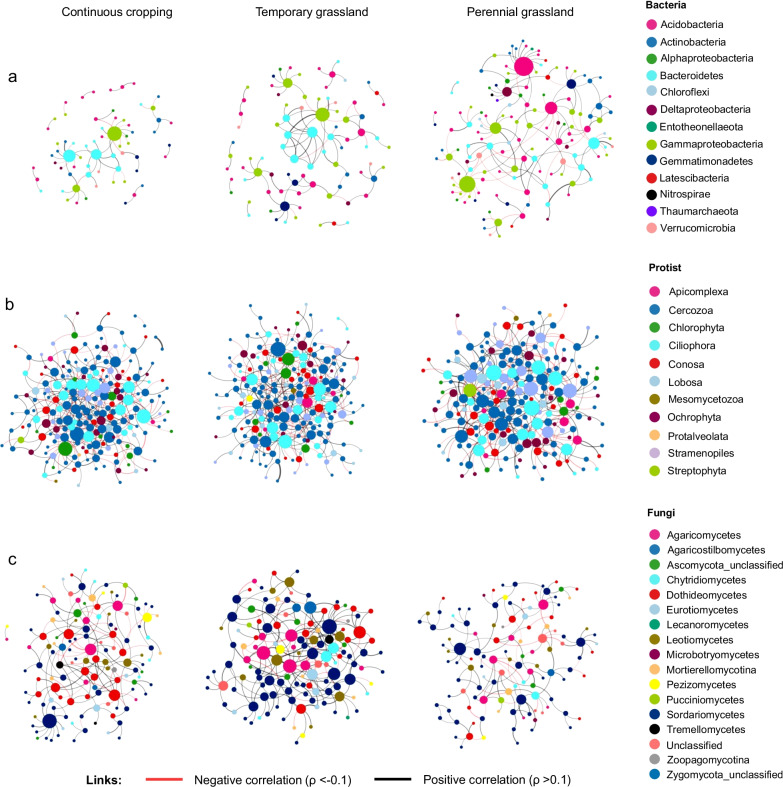
Co-occurrence networks of **a** bacterial, **b** protist and **c** fungal communities in continuous cropping, temporary grassland and perennial grassland. Nodes are colored according to their taxonomic affiliation at phylum and class levels. The size of the nodes is proportional to the number of links per node (i.e. degree). Link thickness is proportional to partial correlations between nodes and represents associative (black, ρ > 0.1) or exclusionary relationships (red, ρ < − 0.1)

We inferred microbial association networks from continuous cropping, temporary and perennial grassland soil samples for the bacterial, protist and fungal communities using a recently developed sparse multivariate Poisson log-normal model [67]. The complexity of networks was estimated using network size (*i.e.* the number of nodes), links, the positive to negative links ratio in the networks, the clustering coefficient (*i.e.* the degree to which nodes are clustered) and the average degree (*i.e.* average links per node) (Table 1). In the bacterial networks, both the number of nodes and links gradually decreased along the land-use intensity gradient (Fig. 4a, Table 1). However, the highest ratio of positive to negative links in the temporary grassland was more than three times higher than that in the other land uses (CC = 2.33, TG = 7.45 and PG = 2.22), due to a decrease in negative associations in the temporary grassland with 13% of negative links comparing to 42% and 45% for continuous cropping and perennial grassland, respectively. For protists, the ratio of positive to negative links was also higher in the temporary grassland, whereas the other network properties were similar between land uses (*i.e.* number of nodes, links and average degree) (Fig. 4b and Table 1). The fungal networks showed a different trend with a higher complexity in the temporary grassland (Fig. 4c and Table 1). The fungal network also revealed an increase in the ratio of positive to negative links in the temporary grassland (CC = 3.20, TG = 5.21 and PG = 4.50), similar to bacterial and protist networks. Networks from the temporary grassland of the bacterial, protist and fungal communities exhibited the highest clustering coefficient.

**Table 1:** Properties of microbial co-occurrence networks

Microbial group	Bacteria			Protist			Fungi			All		
Land use	CC	TG	PG	CC	TG	PG	CC	TG	PG	CC	TG	PG
Total number of OTUs	472	472	472	341	341	341	218	218	218	1031	1031	1031
Nodes	65	85	137	210	208	207	128	132	117	445	517	586
Links	60	93	161	326	330	315	168	211	143	778	971	985
Positive links	42	82	111	182	217	189	128	177	117	519	700	676
Negative links	18	11	50	144	113	126	40	34	26	259	271	309
Ratio ±	2.33	7.45	2.22	1.26	1.92	1.50	3.20	5.21	4.50	2.00	2.58	2.19
Avg. clustering coefficient	0.032	0.105	0.067	0.020	0.037	0.024	0.047	0.094	0.057	0.039	0.049	0.031
Avg. degree	1.85	2.19	2.35	3.10	3.17	3.04	2.63	3.20	2.44	3.50	3.76	3.36

***Associations across microbial groups differ among land uses***

**Fig. 5:**
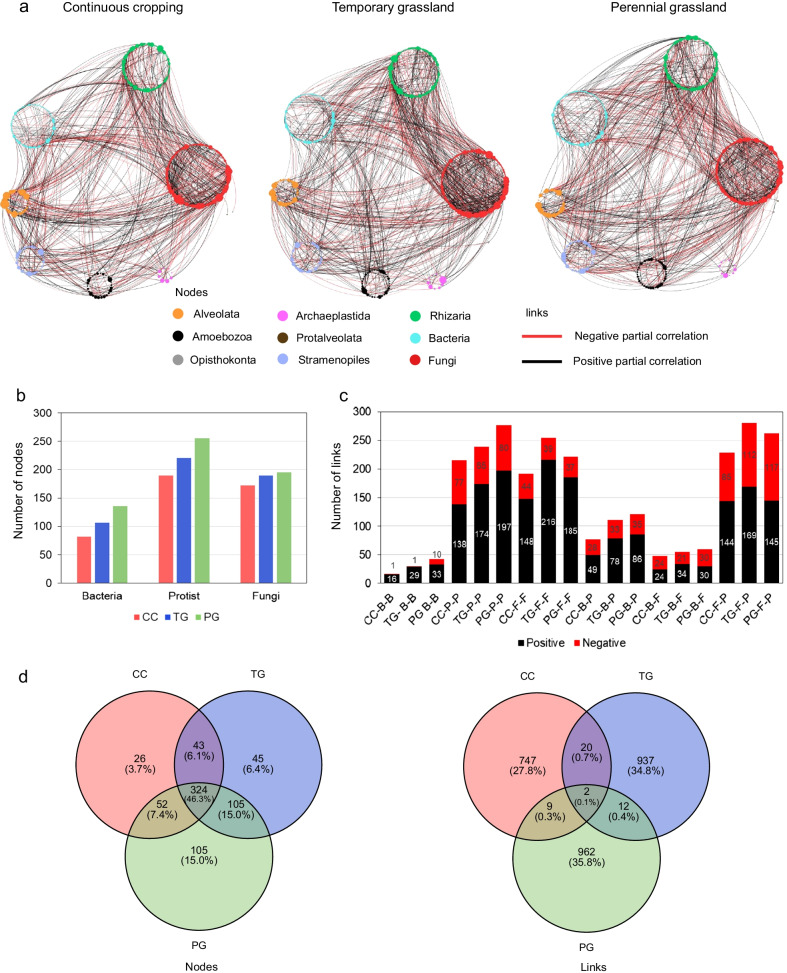
**a** Co-occurrence networks across three groups in continuous cropping (CC), temporary grassland (TG) and perennial grassland (PG). Nodes are colored according to their taxonomic affiliation at phylum levels. The size of the nodes is proportional to the number of links per node (i.e. degree). Link thickness is proportional to partial correlations between nodes and represents associative (black, ρ > 0.08) or exclusionary relationships (red, ρ < -0.08). **b** Number of nodes per land-use for each microbial group. **c** Number of positive (black) and negative (red) links in inter-domain networks for each land-use type and within microbial domains. **d** The Venn Diagrams show the number of shared/unique nodes and links across inter-domains networks

To better understand how land use influences associations between bacteria, protist and fungi, we inferred co-occurrence networks including all three groups for each land use (Fig. 5a). The microbial network with all groups from the perennial grassland was the most complex with 586 nodes and 985 links, followed by the temporary grassland with 517 nodes and 971 links and continuous cropping network with 445 nodes and 778 links (Fig. 5b). By contrast, we found a higher clustering coefficient and average degree in the temporary grassland network indicating that the nodes were more connected than those of continuous cropping and perennial grassland (Table 1). The comparison between inter-group networks showed that 46.3% of nodes are shared between land uses. In contrast, only two microbial links (fungi-fungi and protist-protist) were shared between to the networks from the three land uses (Fig. 5d).

To distinguish differences in taxa co-occurring among land uses, we compared the number of positive and negative links within and between microbial groups (Fig. 5c). Regardless of land use, microbial networks were dominated by fungi-protist (28%), protist-protist (27%) and fungi-fungi (24%) associations. Bacteria-protist and bacteria-bacteria associations represented only 11 and 3% of the total number of links, respectively. However, the positive to negative link ratio for the bacteria-bacteria associations was the highest in temporary grassland (CC = 16, TG = 29 and PG = 3.3). Associations across all three groups were mainly characterized by a higher number of links in temporary and perennial grasslands. This was the case for bacteria-Rhizaria, fungi-Rhizaria, fungi-Alveolata and fungi-Stramenopiles associations (Additional file 1: Figure S6).

## *Discussion*

Investigations of belowground effects of land-use changes have often focused either on the bacterial or on the fungal community. However, as the main consumers of bacteria, protists influence the composition of the soil microbiome [26, 70]. This highlights the need to adopt more comprehensive approaches for holistic insights into the soil microbiome [24]. Here, we performed comparative and integrative analyses of the soil bacterial, fungal and protistan communities across a gradient of land-use intensity based on cropping frequency. We showed that *β*-diversity of all three domains was affected by land-use type, with significantly different communities in the continuous cropping, temporary grassland and perennial grassland. Little is known about the importance of land-use intensity in driving soil protists and there are large discrepancies between studies [27]. Although climatic factors have been described as the top predictor of composition of protist communities [71], our result indicate that protists can be affected by changes in land use to the same extent as bacterial and fungal communities. However, the *α*-diversity of the protist and fungal communities showed no significant differences across land uses, although the bacterial *α*-diversity did, with the highest diversity in continuous cropping and temporary grassland compared to perennial grassland. In line with our results, previous studies found that shifts to agricultural land-use increased bacterial diversity [72, 73]. This might be explained by the rotation of plant communities in these systems that likely led to a greater diversity in soil nutrients through root exudates and crop residues, ultimately increasing bacterial diversity [74]. This is congruent with the observed differences in the biochemical nature of the soil organic matter (SOM) between the long-term grasslands and continuous cropping systems studied here [75]. The relative abundances of N-cycling communities (*i.e.* nitrifiers, denitrifiers and DNRA bacteria) were the highest in the continuous cropping, suggesting that land-use can be a driver for these functional groups. Accordingly, previous studies reported significant effects of land use on the relative abundances of AOB and DNRA bacteria [55, 76]. However, since microbial communities involved in N-cycling processes resulting in nitrogen losses (denitrification and nitrification) as well as in nitrogen retention (DNRA) were enriched in the crop rotation, further work is needed to determine the fate of inorganic N in these systems.

To determine potential legacy effects of prior land use (i.e. continuous cropping followed by three years of temporary grassland), the soil samples were collected in all land uses when the cycle of the temporary grassland ended (TG). Without any effect of land-use history, it was expected that microbiomes would be more similar under temporary and perennial grasslands than under continuous cropping. Instead, the smallest differences in community structure for all domains were observed between temporary grassland and continuous cropping, which suggests lasting effects of the cropping system preceding the temporary grasslands. The impact of land-use history on microbial communities has been investigated in previous studies with large variation in the persistence of the legacy effects [77, 78]. For example, past arable farming land resulted in long-lasting legacies on forest microbial communities that were persisting over half a century after agricultural abandonment [13, 79]. An explanation for these long-lasting effects is that historical land use caused shifts in soil properties, such as C and N content or pH, which are important drivers of soil microbial communities and may require decades to recover. Consistent with this and our results, Crème et al. (2018) [75] observed that despite the insertion of three years of temporary grasslands within the crop rotation, the biogeochemical signature of the soil organic matter was more similar to the continuous cropping system than to the permanent grassland. Moreover, plant species can also affect differently soil microbial communities through a myriad of processes [8, 80] and create legacies that are detectable under subsequent plant communities [81], therefore also explaining the similarities between temporary grassland and continuous cropping [82]. Overall, we found that prior cropping systems that were at least three-year-old continued to play a role in shaping microbial community composition across groups, which highlight the importance to consider legacies of past land-use when assessing the impact of management practices. This is all the more important considering that such changes in soil communities can in turn affect subsequent plant communities [83].

Due to intrinsic differences in eco-evolutionary dynamics between perennial and annual systems, it can be hypothesized that plant-soil feedbacks can lead to more complex and more connected networks in permanent grassland. However, when inferring microbial co-occurrence networks for each group (i.e. bacteria, protist and fungi), only the bacterial network was more complex in the perennial grassland with a gradual increase in the number of nodes and links between bacterial OTUs along with decreasing land-use intensity (i.e. from CC to PG). This is in agreement with a previous study showing higher bacterial network complexity in grassland than cropping system [84]. Interestingly, perennial grasslands also had the lowest bacterial diversity, which suggests that changes in soil microbial diversity do not necessarily reflect changes in microbial networks [85]. In contrast to a previous study showing that agricultural intensification reduces fungal network complexity [17], we found a higher complexity in the temporary grassland compared to the perennial and continuous cropping systems. This discrepancy may be attributed to the fact that root-associated fungi were monitored by Banerjee et al. [17] whereas the present study focuses on soil communities. Differences in the structure and composition of the protist community across land-use types were not mirrored in the network complexity. However, when considering the link type, we found an increase in the positive to negative link ratio in the temporary grasslands for protists as well as the other two groups, indicating that positive associations between soil microorganisms were promoted. This pattern might be due to increased niche differentiation when alternating temporary grassland and crop rotation, which results in a decrease of competition between microbial species [86, 87]. On the opposite, the decrease in the positive to negative link ratio in the cropping systems suggests less effective establishment of cooperation rather than increased competition between microbial taxa. Alternatively, previous work showed that differences in community evenness are likely to also affect the positive edge percentage since less prevalent taxa tend to contribute more to negative edges [32]. In any case, further studies are required to decipher whether these changes in co-occurrence networks were induced by biotic interactions or differences in environmental filters among the three land-use types.

Finally, we inferred networks encompassing all three groups to obtain an integrated and holistic view of the soil microbiome along the land-use intensity gradient. Similar to the bacterial network, we found a higher complexity of the inter-group network under permanent grasslands, which supports our hypothesis that perennial systems lead to more connected microbial networks. This increase in complexity was mostly due to an increase in the number of bacterial and protist nodes as well as bacteria-bacteria, protist-protist and bacteria-protist links, despite that the individual protist networks were similar between land uses in contrast to those of the bacterial. While almost half of the nodes of the inter-domain networks were similar, links were distinct between land-use types, indicating that microbial associations are more sensitive than community structure to land-use intensification. That land-use management affected associations within the soil microbiome is important to consider given that recent studies have shown that ecosystem functioning could be related to microbial network complexity [33, 88]. Whatever the land-use, protists were dominant in the inter-group network (Fig. 5b), especially the Rhizaria (Cercozoa), which were highly connected to both bacteria and fungi. Although co-occurrence networks suffer of spurious correlations when the effects of habitat filtering are strong, they can also recapitulate possible interactions between microorganisms under certain conditions [89, 90]. While not all protists feed on other organisms, they are known as the main consumers of bacteria [29]. However, we found more negative associations between protists (Rhizaria but also Amoebozoa and Stramenopiles) and fungi than between protists and bacteria. Accordingly, recent work suggest that protist feeding on fungi might equally be important [91]. On the other hand, some fungi have developed trapping structures, such as adhesive spores, hyphae to capture soil-inhabiting microorganisms such as protists [92]. Fungi can also be protist parasites, when for example amoeba ingests the spores or conidia, resulting in its death [93]. These predatory/parasitic interactions could explain some of negative associations between fungi and protists observed in our study.

## Conclusions

Using a holistic microbiome investigation of bacterial, fungal and protist communities in a long-term field experiment managed under different levels of land use intensity, we showed that land management only affected *α*-diversity of the bacterial community, with increased diversity in the continuous cropping system. However, we identified a clear shift in the structure and the composition of all communities in response to land use, in particular between the continuous cropping and the perennial grassland. Moreover, our results showed legacy effects of cropping on the structure of the soil microbiome that lasted after three years of temporary grasslands. This highlights that prior land use can shape the present-day community for multiple microbial groups across domains. The perennial grassland system led to more complex bacterial as well as inter-domain networks, which can have implication for the contribution of microbes to ecosystem multifunctionality [16]. Inter-domain networks also revealed the predominant role of the protist as key taxa in soil microbiome networks across all land-use types. Future work need to validate the importance of protists in shaping soil microbial communities, directly through biotic interactions and/or indirectly through changes in abiotic factors.

## Supplementary Information

**Additional file 1: Table S1.** Total carbon and nitrogen in continuous cropping (CC), temporary grassland (TG) and perennial grassland (PG). **Figure S1.** Experimental design of the long-term observatory near Lusignan, France. (a) The experiment is set up as a randomized complete block design divided in four blocks comprising (b) a three year crop rotation of maize-wheat-barley (CC), a three-year temporary grassland alternated with the three-year crop rotation (TG) and a permanent grassland (PG). **Figure S2**. *α*-diversity of the microbial communities in continuous cropping (CC), temporary grassland (TG) and perennial grassland (PG). (a) Simpson’s reciprocal, (b) Shannon, (c) observed species and (d) Faith’s phylogenetic diversity (PD) indices are shown. Different letters above the boxplots indicate significant differences according to Tukey’s test (p-value < 0.05). **Figure S3.** Bray–Curtis distances between land uses (mean ± s.d.). Different letters above the bars indicate significant differences according to Tukey’s test (p-value < 0.05). **Figure S4.** Microbial composition in continuous cropping (CC), temporary grassland (TG) and perennial grassland (PG) of bacterial (a), protist (b) and fungal (c) communities. Relative abundances are shown at the phylum and class levels and expressed as a percentage of the total number of OTUs. **Figure S5.** Ternary plots representing the composition of the microbial community under different land uses. The distributions of the most abundant OTUs in the bacterial (a), protist (b) and fungal (c) communities are shown. The position of each circle on the axis represents the contribution of the indicated land use to the relative abundance of each OTU. The size of the circle indicates the mean frequencies of each OTU in all samples. The colors indicate the affiliation of OTUs at the phylum or class levels. **Figure S6.** Number of positive (black) and negative (red) links between bacteria (B) or fungi (F) and groups of protists.

**Additional file 2: Script 1.** R code for differential abundance analysis using DESeq2 package. **Script 2.** R code of network analysis using a sparse multivariate Poisson log-normal (PLN) model.

## Data Availability

Raw sequences were deposited at the NCBI under the accession numbers PRJNA741976, PRJNA741982 and PRJNA742156. Data that support the findings of this study is also available from the corresponding author upon reasonable request.

## References

[1] Sala OE, Chapin FS, Armesto JJ, Berlow E, Bloomfield J, Dirzo R (2000). Biodiversity—global biodiversity scenarios for the year 2100. Science.

[2] Zabel F, Delzeit R, Schneider JM, Seppelt R, Mauser W, Vaclavik T. Global impacts of future cropland expansion and intensification on agricultural markets and biodiversity. Nat Commun. 2019;10. 10.1038/s41467-019-10775-z PMC6598988 31253787

[3] Allan E, Manning P, Alt F, Binkenstein J, Blaser S, Bluethgen N (2015). Land use intensification alters ecosystem multifunctionality via loss of biodiversity and changes to functional composition. Ecol Lett.

[4] Burney JA, Davis SJ, Lobell DB (2010). Greenhouse gas mitigation by agricultural intensification. PNAS.

[5] Cameron KC, Di HJ, Moir JL (2013). Nitrogen losses from the soil/plant system: a review. Ann Appl Biol.

[6] De Deyn GB, Van der Putten WH (2005). Linking aboveground and belowground diversity. Trends Ecol Evol.

[7] Wardle DA, Bardgett RD, Klironomos JN, Setala H, van der Putten WH, Wall DH (2004). Ecological linkages between aboveground and belowground biota. Science.

[8] Philippot L, Raaijmakers JM, Lemanceau P, van der Putten WH (2013). Going back to the roots: the microbial ecology of the rhizosphere. Nat Rev Microbiol.

[9] Hartmann M, Frey B, Mayer J, Mader P, Widmer F (2015). Distinct soil microbial diversity under long-term organic and conventional farming. ISME J.

[10] Lauber CL, Strickland MS, Bradford MA, Fierer N (2008). The influence of soil properties on the structure of bacterial and fungal communities across land-use types. Soil Biol Biochem.

[11] Leff JW, Jones SE, Prober SM, Barberan A, Borer ET, Firn JL (2015). Consistent responses of soil microbial communities to elevated nutrient inputs in grasslands across the globe. PNAS.

[12] Li XG, Jousset A, de Boer W, Carrion VJ, Zhang TL, Wang XX (2019). Legacy of land use history determines reprogramming of plant physiology by soil microbiome. ISME J.

[13] Turley NE, Bell-Dereske L, Evans SE, Brudvig LA (2020). Agricultural land-use history and restoration impact soil microbial biodiversity. J Appl Ecol.

[14] Philippot L, Spor A, Henault C, Bru D, Bizouard F, Jones CM (2013). Loss in microbial diversity affects nitrogen cycling in soil. ISME J.

[15] van der Heijden MGA, Bardgett RD, van Straalen NM (2008). The unseen majority: soil microbes as drivers of plant diversity and productivity in terrestrial ecosystems. Ecol Lett.

[16] Wagg C, Bender SF, Widmer F, van der Heijden MGA (2014). Soil biodiversity and soil community composition determine ecosystem multifunctionality. PNAS.

[17] Banerjee S, Walder F, Buchi L, Meyer M, Held AY, Gattinger A (2019). Agricultural intensification reduces microbial network complexity and the abundance of keystone taxa in roots. ISME J.

[18] Estendorfer J, Stempfhuber B, Haury P, Vestergaard G, Rillig MC, Joshi J, et al. The influence of land use intensity on the plant-associated microbiome of *Dactylis glomerata* L. Front Plant Sci. 2017;8. 10.3389/fpls.2017.00930 PMC5478725 28680426

[19] Hartman K, van der Heijden MGA, Wittwer RA, Banerjee S, Walser JC, Schlaeppi K. Cropping practices manipulate abundance patterns of root and soil microbiome members paving the way to smart farming. Microbiome. 2018;6. 10.1186/s40168-017-0389-9 PMC5771023 29338764

[20] Tardy V, Spor A, Mathieu O, Leveque J, Terrat S, Plassart P (2015). Shifts in microbial diversity through land use intensity as drivers of carbon mineralization in soil. Soil Biol Biochem.

[21] de Vos MGJ, Zagorski M, McNally A, Bollenbach T (2017). Interaction networks, ecological stability, and collective antibiotic tolerance in polymicrobial infections. PNAS.

[22] Faust K, Raes J (2012). Microbial interactions: from networks to models. Nat Rev Microbiol.

[23] Friedman J, Higgins LM, Gore J. Community structure follows simple assembly rules in microbial microcosms. Nat Ecol Evol. 2017;1. 10.1038/s41559-017-0109 28812687

[24] Geisen S, Briones MJI, Gan HJ, Behan-Pelletier VM, Friman VP, de Groot GA, et al. A methodological framework to embrace soil biodiversity. Soil Biol Biochem. 2019;136.

[25] Russel J, Roder HL, Madsen JS, Burmolle M, Sorensen SJ (2017). Antagonism correlates with metabolic similarity in diverse bacteria. PNAS.

[26] Gao ZL, Karlsson I, Geisen S, Kowalchuk G, Jousset A (2019). Protists: puppet masters of the rhizosphere microbiome. Trends Plant Sci.

[27] Geisen S, Mitchell EAD, Adl S, Bonkowski M, Dunthorn M, Ekelund F (2018). Soil protists: a fertile frontier in soil biology research. FEMS Microbiol Rev.

[28] Xiong W, Song YQ, Yang KM, Gu Y, Wei Z, Kowalchuk GA, et al. Rhizosphere protists are key determinants of plant health. Microbiome. 2020;8. 10.1186/s40168-020-00799-9 PMC7055055 32127034

[29] Bonkowski M (2004). Protozoa and plant growth: the microbial loop in soil revisited. New Phytol.

[30] Clarholm M (1985). Interactions of bacteria, protozoa and plants leading to mineralization of soil-nitrogen. Soil Biol Biochem.

[31] Barberan A, Bates ST, Casamayor EO, Fierer N (2012). Using network analysis to explore co-occurrence patterns in soil microbial communities. ISME J.

[32] Faust K, Lima-Mendez G, Lerat JS, Sathirapongsasuti JF, Knight R, Huttenhower C, et al. Cross-biome comparison of microbial association networks. Front Microbiol. 2015;6. 10.3389/fmicb.2015.01200 PMC4621437 26579106

[33] Wagg C, Schlaeppi K, Banerjee S, Kuramae EE, van der Heijden MGA. Fungal-bacterial diversity and microbiome complexity predict ecosystem functioning. Nat Commun. 2019;10. 10.1038/s41467-019-12798-y PMC6813331 31649246

[34] LeBauer DS, Treseder KK (2008). Nitrogen limitation of net primary productivity in terrestrial ecosystems is globally distributed. Ecology.

[35] Hu T, Chabbi A. Does the higher root carbon contribution to soil under cropping cycles following grassland conversion also increase shoot biomass? Sci Total Environ. 2021;752. 10.1016/j.scitotenv.2020.141684 32892038

[36] Lemaire G, Jeuffroy MH, Gastal F (2008). Diagnosis tool for plant and crop N status in vegetative stage theory and practices for crop N management. Eur J Agron.

[37] Berry D, Ben Mahfoudh K, Wagner M, Loy A (2011). Barcoded primers used in multiplex amplicon pyrosequencing bias amplification. Appl Environ Microbiol.

[38] Bower SM, Carnegie RB, Goh B, Jones SRM, Lowe GJ, Mak MWS (2004). Preferential PCR amplification of parasitic protistan small subunit rDNA from metazoan tissues. J Eukaryot Microbiol.

[39] Simon M, Jardillier L, Deschamps P, Moreira D, Restoux G, Bertolino P (2015). Complex communities of small protists and unexpected occurrence of typical marine lineages in shallow freshwater systems. Environ Microbiol.

[40] Gardes M, Bruns TD (1993). ITS Primers with enhanced specificity for basidiomycetes—application to the identification of mycorrhizae and rusts. Mol Ecol.

[41] White TJ, Bruns TD, Lee SB, Taylor JW, Innis MA, Gelfand DH, Sninsky JJ, White TJ (1990). Amplification and direct sequencing of fungal ribosomal RNA genes for phylogenetics. PCR-protocols and applications: a laboratory manual.

[42] Zhang JJ, Kobert K, Flouri T, Stamatakis A (2014). PEAR: a fast and accurate illumina paired-end reAd mergeR. Bioinformatics.

[43] Caporaso JG, Kuczynski J, Stombaugh J, Bittinger K, Bushman FD, Costello EK (2010). QIIME allows analysis of high-throughput community sequencing data. Nat Methods.

[44] Rognes T, Flouri T, Nichols B, Quince C, Mahe F. VSEARCH: a versatile open source tool for metagenomics. Peerj. 2016;4. 10.7717/peerj.2584 PMC5075697 27781170

[45] Engelhardt IC, Welty A, Blazewicz SJ, Bru D, Rouard N, Breuil MC (2018). Depth matters: effects of precipitation regime on soil microbial activity upon rewetting of a plant-soil system. ISME J.

[46] Caporaso JG, Bittinger K, Bushman FD, DeSantis TZ, Andersen GL, Knight R (2010). PyNAST: a flexible tool for aligning sequences to a template alignment. Bioinformatics.

[47] Price MN, Dehal PS, Arkin AP. FastTree 2-approximately maximum-likelihood trees for large alignments. Plos One. 2010;5. 10.1371/journal.pone.0009490 PMC2835736 20224823

[48] Edgar RC (2010). Search and clustering orders of magnitude faster than BLAST. Bioinformatics.

[49] Quast C, Pruesse E, Yilmaz P, Gerken J, Schweer T, Yarza P (2013). The SILVA ribosomal RNA gene database project: improved data processing and web-based tools. Nucleic Acids Res.

[50] Guillou L, Bachar D, Audic S, Bass D, Berney C, Bittner L (2013). The Protist Ribosomal Reference database (PR2): a catalog of unicellular eukaryote Small Sub-Unit rRNA sequences with curated taxonomy. Nucleic Acids Res.

[51] Altschul SF, Gish W, Miller W, Myers EW, Lipman DJ (1990). Basic local alignment search tool. J Mol Biol.

[52] Abarenkov K, Nilsson RH, Larsson KH, Alexander IJ, Eberhardt U, Erland S (2010). The UNITE database for molecular identification of fungi - recent updates and future perspectives. New Phytol.

[53] Faith DP (1992). Conservation evaluation and phylogenetic diversity. Biol Conserv.

[54] Muyzer G, Dewaal EC, Uitterlinden AG (1993). Profiling of complex microbial populations by denaturing gradient gel electrophoresis analysis of polymerase chain reaction-amplified genes coding for 16S rRNA. Appl Environ Microbiol.

[55] Bru D, Ramette A, Saby NPA, Dequiedt S, Ranjard L, Jolivet C (2011). Determinants of the distribution of nitrogen-cycling microbial communities at the landscape scale. ISME J.

[56] Leininger S, Urich T, Schloter M, Schwark L, Qi J, Nicol GW (2006). Archaea predominate among ammonia-oxidizing prokaryotes in soils. Nature.

[57] Poly F, Monrozier LJ, Bally R (2001). Improvement in the RFLP procedure for studying the diversity of nifH genes in communities of nitrogen fixers in soil. Res Microbiol.

[58] Mohan SB, Schmid M, Jetten M, Cole J (2004). Detection and widespread distribution of the nrfA gene encoding nitrite reduction to ammonia, a short circuit in the biological nitrogen cycle that competes with denitrification. FEMS Microbiol Ecol.

[59] Welsh A, Chee-Sanford JC, Connor LM, Loffler FE, Sanford RA (2014). Refined NrfA phylogeny improves PCR-based nrfA gene detection. Appl Environ Microbiol.

[60] R Core Team. R: A language and environment for statistical computing. Vienna: R Foundation for Statistical Computing; 2020.

[61] de Mendiburu F. Agricolae: Statistical Procedures for Agricultural Research. R package version 1.2-8. 2017.

[62] Oksanen. J, Blanchet FG, Friendly M, Kindt R, Legendre P, McGlinn D, et al. vegan: community ecology package 2.5.7. 2018.

[63] Martinez Arbizu P. pairwiseAdonis: Pairwise multilevel comparison using adonis. R package version 0.4. 2019.

[64] Love MI, Huber W, Anders S. Moderated estimation of fold change and dispersion for RNA-seq data with DESeq2. Genome Biol. 2014;15. 10.1186/s13059-014-0550-8 PMC4302049 25516281

[65] Hamilton NE, Ferry M (2018). ggtern: ternary diagrams using ggplot2. J Stat Softw.

[66] Conway JR, Lex A, Gehlenborg N (2017). UpSetR: an R package for the visualization of intersecting sets and their properties. Bioinformatics.

[67] Chiquet J, Mariadassou M, S. R. Variational inference for sparse network reconstruction from count data. ICML*.* 2018; 97: 1162–1171.

[68] Liu H, Roeder K, Wasserman L (2010). Stability approach to regularization selection (StARS) for high dimensional graphical models. Adv Neural Inf Process Syst.

[69] Shannon P, Markiel A, Ozier O, Baliga NS, Wang JT, Ramage D (2003). Cytoscape: a software environment for integrated models of biomolecular interaction networks. Genome Res.

[70] Geisen S (2016). The bacterial-fungal energy channel concept challenged by enormous functional versatility of soil protists. Soil Biol Biochem.

[71] Oliverio AM, Geisen S, Delgado-Baquerizo M, Maestre FT, Turner BL, Fierer N. The global-scale distributions of soil protists and their contributions to belowground systems. Sci Adv. 2020;6. 10.1126/sciadv.aax8787 PMC6981079 32042898

[72] Delgado-Baquerizo M, Maestre FT, Reich PB, Jeffries TC, Gaitan JJ, Encinar D, et al. Microbial diversity drives multifunctionality in terrestrial ecosystems. Nat Commun. 2016;7. 10.1038/ncomms10541 PMC4738359 26817514

[73] Rodrigues JLM, Pellizari VH, Mueller R, Baek K, Jesus ED, Paula FS (2013). Conversion of the Amazon rainforest to agriculture results in biotic homogenization of soil bacterial communities. PNAS.

[74] Tiemann LK, Grandy AS, Atkinson EE, Marin-Spiotta E, McDaniel MD (2015). Crop rotational diversity enhances belowground communities and functions in an agroecosystem. Ecol Lett.

[75] Creme A, Rumpel C, Le Roux X, Romian A, Lan T, Chabbi A (2018). Ley grassland under temperate climate had a legacy effect on soil organic matter quantity, biogeochemical signature and microbial activities. Soil Biol Biochem.

[76] Putz M, Schleusner P, Rutting T, Hallin S (2018). Relative abundance of denitrifying and DNRA bacteria and their activity determine nitrogen retention or loss in agricultural soil. Soil Biol Biochem.

[77] Buckley DH, Schmidt TM (2001). The structure of microbial communities in soil and the lasting impact of cultivation. Microb Ecol.

[78] Jangid K, Williams MA, Franzluebbers AJ, Schmidt TM, Coleman DC, Whitman WB (2011). Land-use history has a stronger impact on soil microbial community composition than aboveground vegetation and soil properties. Soil Biol Biochem.

[79] Fichtner A, von Oheimb G, Hardtle W, Wilken C, Gutknecht JLM (2014). Effects of anthropogenic disturbances on soil microbial communities in oak forests persist for more than 100 years. Soil Biol Biochem.

[80] Moreau D, Bardgett RD, Finlay RD, Jones DL, Philippot L (2019). A plant perspective on nitrogen cycling in the rhizosphere. Funct Ecol.

[81] Hannula SE, Heinen R, Huberty M, Steinauer K, De Long JR, Jongen R (2021). Persistence of plant-mediated microbial soil legacy effects in soil and inside roots. Nat Commun.

[82] Heinen R, Hannula SE, De Long JR, Huberty M, Jongen R, Kielak A (2020). Plant community composition steers grassland vegetation via soil legacy effects. Ecol Lett.

[83] van der Putten WH, Bardgett RD, Bever JD, Bezemer TM, Casper BB, Fukami T (2013). Plant-soil feedbacks: the past, the present and future challenges. J Ecol.

[84] Karimi B, Dequiedt S, Terrat S, Jolivet C, Arrouays D, Wincker P, et al. Biogeography of soil bacterial networks along a gradient of cropping intensity. Sci Rep. 2019;9. 10.1038/s41598-019-40422-y PMC6405751 30846759

[85] Shi SJ, Nuccio EE, Shi ZJ, He ZL, Zhou JZ, Firestone MK (2016). The interconnected rhizosphere: high network complexity dominates rhizosphere assemblages. Ecol Lett.

[86] Lupatini M, Suleiman AKA, Jacques RJS, Antoniolli ZI, Ferreira AdS, Kuramae EE et al Network topology reveals high connectance levels and few key microbial genera within soils. Front Environ Sci. 2014;8.

[87] Schlatter DC, Bakker MG, Bradeen JM, Kinkel LL (2015). Plant community richness and microbial interactions structure bacterial communities in soil. Ecology.

[88] Morrien E, Hannula SE, Snoek LB, Helmsing NR, Zweers H, de Hollander M, et al. Soil networks become more connected and take up more carbon as nature restoration progresses. Nat Commun. 2017;8. 10.1038/ncomms14349 PMC5309817 28176768

[89] Berry D, Widder S. Deciphering microbial interactions and detecting keystone species with co-occurrence networks. Front Microbiol. 2014;5. 10.3389/fmicb.2014.00219 PMC4033041 24904535

[90] Zhao ZB, He JZ, Geisen S, Han LL, Wang JT, Shen JP, et al. Protist communities are more sensitive to nitrogen fertilization than other microorganisms in diverse agricultural soils. Microbiome. 2019;7. 10.1186/s40168-019-0647-0 PMC6393985 30813951

[91] Geisen S, Koller R, Hunninghaus M, Dumack K, Urich T, Bonkowski M (2016). The soil food web revisited: diverse and widespread mycophagous soil protists. Soil Biol Biochem.

[92] Corsaro D, Kohsler M, Wylezich C, Venditti D, Walochnik J, Michel R (2018). New insights from molecular phylogenetics of amoebophagous fungi (Zoopagomycota, Zoopagales). Parasitol Res.

[93] Scheid PL (2018). Amoebophagous fungi as predators and parasites of potentially pathogenic free-living amoebae. Open Parasitol J.

