# Supplementary material for: Land-use intensification differentially affects bacterial, fungal and protist communities and decreases microbiome network complexity
Source: Environ Microbiome. 2022 Jan 6;17:1. doi: 10.1186/s40793-021-00396-9 (PMC8740439; doi:10.1186/s40793-021-00396-9)
Supplement: Supplementary file 1 — Additional file 1: Table S1. Total carbon and nitrogen in continuous cropping (CC), temporary grassland (TG) and perennial grassland (PG). Figure S1. Experimental design of the long-term observatory near Lusignan, France. (a) The experiment is set up as a randomized complete block design divided in four blocks comprising (b) a three year crop rotation of maize-wheat-barley (CC), a three-year temporary grassland alternated with the three-year crop rotation (TG) and a permanent grassland (PG). Figure S2. α-diversity of the microbial communities in continuous cropping (CC), temporary grassland (TG) and perennial grassland (PG). (a) Simpson’s reciprocal, (b) Shannon, (c) observed species and (d) Faith’s phylogenetic diversity (PD) indices are shown. Different letters above the boxplots indicate significant differences according to Tukey’s test (p-value < 0.05). Figure S3. Bray–Curtis distances between land uses (mean ± s.d.). Different letters above the bars indicate significant differences according to Tukey’s test (p-value < 0.05). Figure S4. Microbial composition in continuous cropping (CC), temporary grassland (TG) and perennial grassland (PG) of bacterial (a), protist (b) and fungal (c) communities. Relative abundances are shown at the phylum and class levels and expressed as a percentage of the total number of OTUs. Figure S5. Ternary plots representing the composition of the microbial community under different land uses. The distributions of the most abundant OTUs in the bacterial (a), protist (b) and fungal (c) communities are shown. The position of each circle on the axis represents the contribution of the indicated land use to the relative abundance of each OTU. The size of the circle indicates the mean frequencies of each OTU in all samples. The colors indicate the affiliation of OTUs at the phylum or class levels. Figure S6. Number of positive (black) and negative (red) links between bacteria (B) or fungi (F) and groups of protists. [file 40793_2021_396_MOESM1_ESM.docx]

**Additional File 1**

***Land-use intensification differentially affects bacterial, fungal and protist communities and decreases microbiome network complexity***

Sana Romdhane^1^, Aymé Spor^1^, Samiran Banerjee^2,3^, Marie-Christine Breuil^1^, David Bru^1^, Abad Chabbi^4,5^, Sara Hallin^6^,  Marcel GA van der Heijden^2,7^, Aurélien Saghai^6^, Laurent Philippot^1*^

^1^University Bourgogne Franche Comte, INRAE, AgroSup Dijon, Department of Agroecology, Dijon, France

^2^Agroscope, Plant-Soil Interactions Group, Zurich, Switzerland

^3^Department of Biological Sciences, North Dakota State University, Fargo 58102, USA

^4^ECOSYS, UMR INRAE, AgroParisTech, Thiverval-Grignon, France

^5^CNRS, Institute of Ecology and Environmental Sciences-Paris (iEES-Paris, UMR Sorbonne Université, CNRS, INRAE), Thiverval-Grignon, France

^6^Swedish University of Agricultural Sciences, Department of Forest Mycology and Plant Pathology, Uppsala, Sweden

^7^University of Zurich, Department of Plant and Microbial Biology, Zurich, Switzerland

^*^Corresponding author: Laurent.philippot@inrae.fr

**Table S1.** Total carbon and nitrogen in continuous cropping (CC), temporary grassland (TG) and perennial grassland (PG).

|  | **Carbon (mg g-1)** | **Nitrogen (mg g-1)** | **C/N** |
| --- | --- | --- | --- |
| **CC** | 9.78±1.07 | 1.05±0.09 | 9.33±0.24 |
| **TG** | 10.74±1.15 | 1.13±0.09 | 9.45±0.24 |
| **PG** | 11.13±0.77 | 1.18±0.07 | 9.41±0.14 |

**
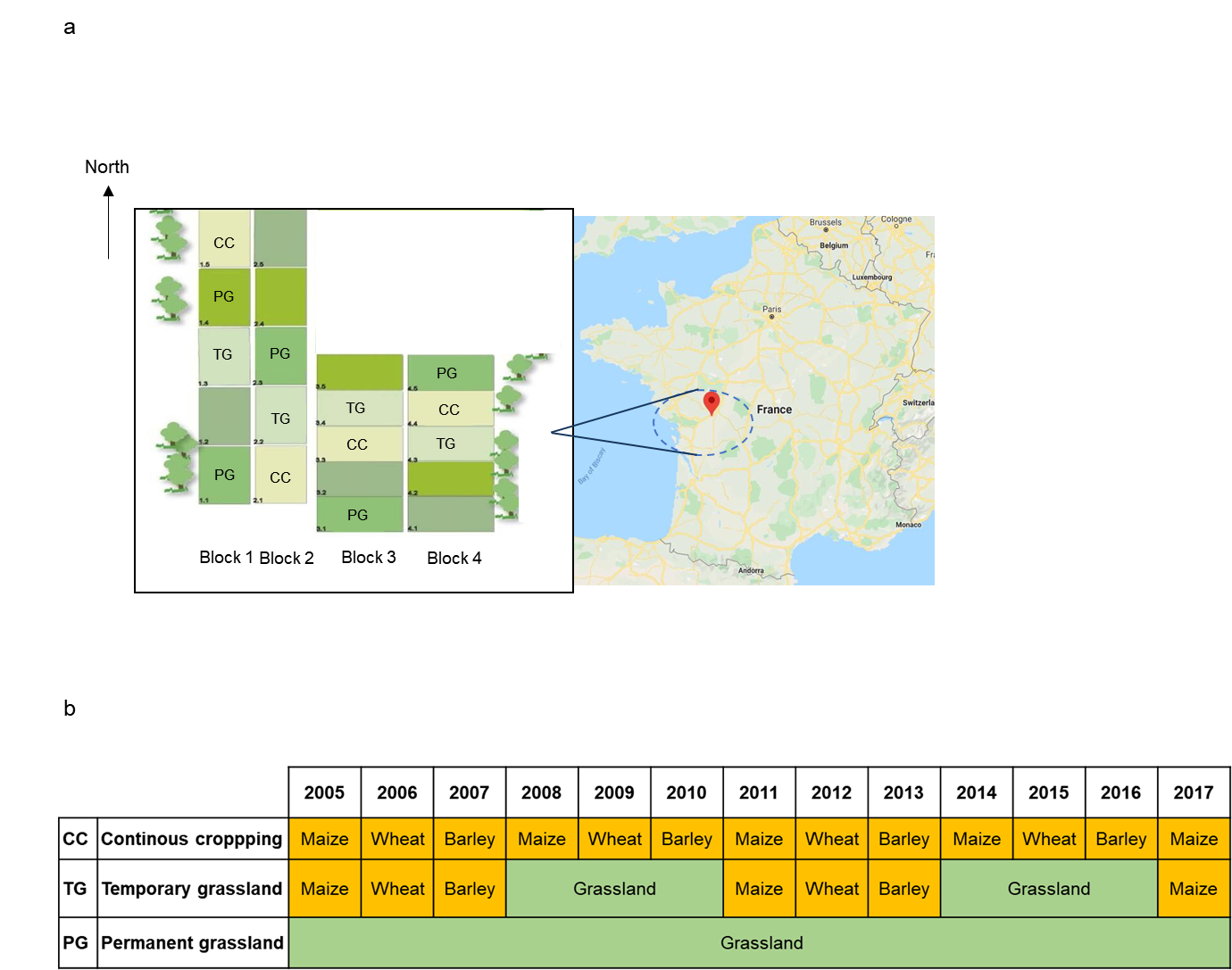
**

**Figure S1.** Experimental design of the long-term observatory near Lusignan, France. (a) The experiment is set up as a randomized complete block design divided in four blocks comprising (b) a three year crop rotation of maize-wheat-barley (CC), a three-year temporary grassland alternated with the three-year crop rotation (TG) and a permanent grassland (PG).

**
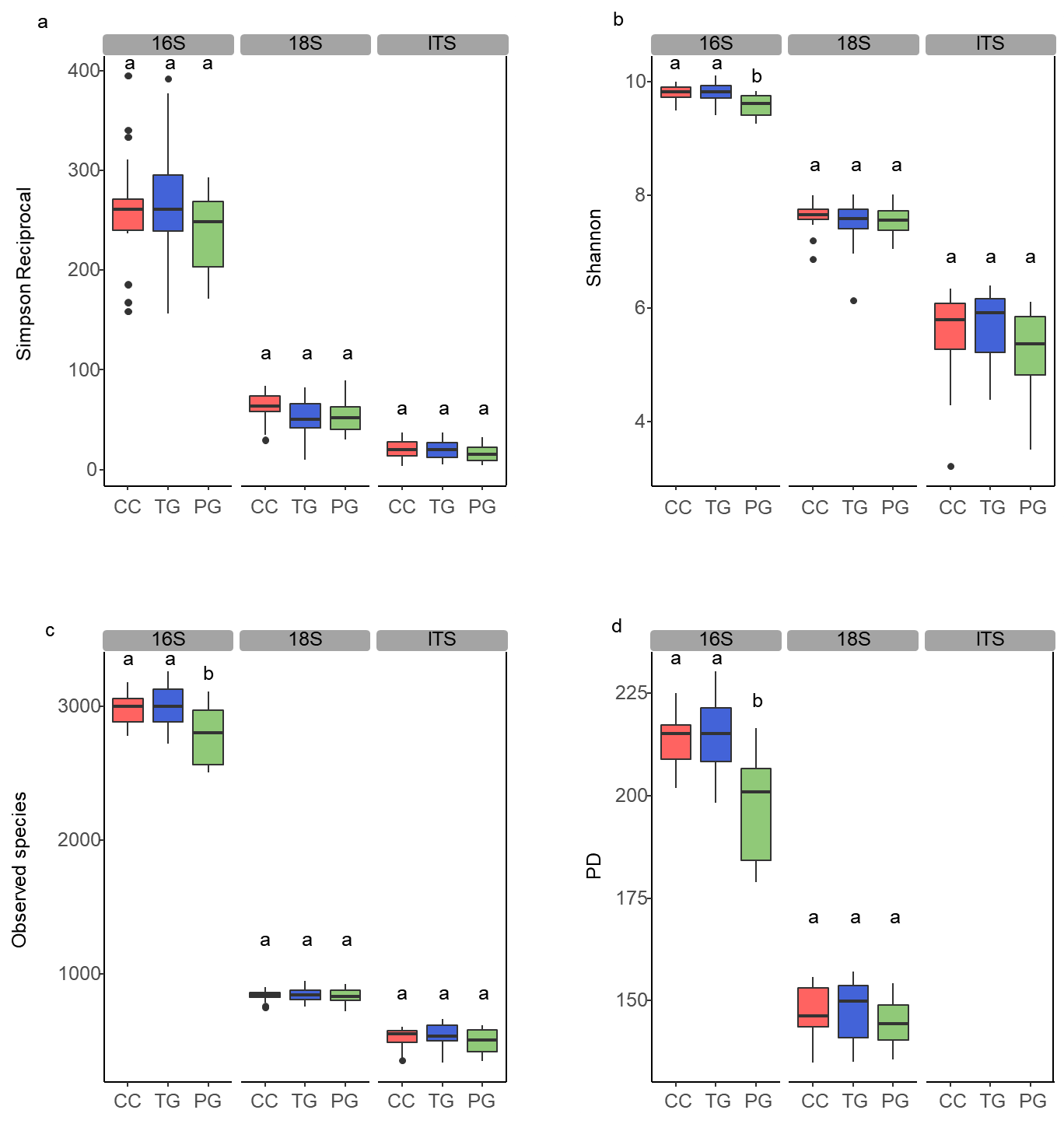
**

**Figure S2**. *α*-diversity of the microbial communities in continuous cropping (CC), temporary grassland (TG) and perennial grassland (PG). (a) Simpson’s reciprocal, (b) Shannon, (c) observed species and (d) Faith’s phylogenetic diversity (PD) indices are shown. Different letters above the boxplots indicate significant differences according to Tukey’s test (p-value < 0.05).

**
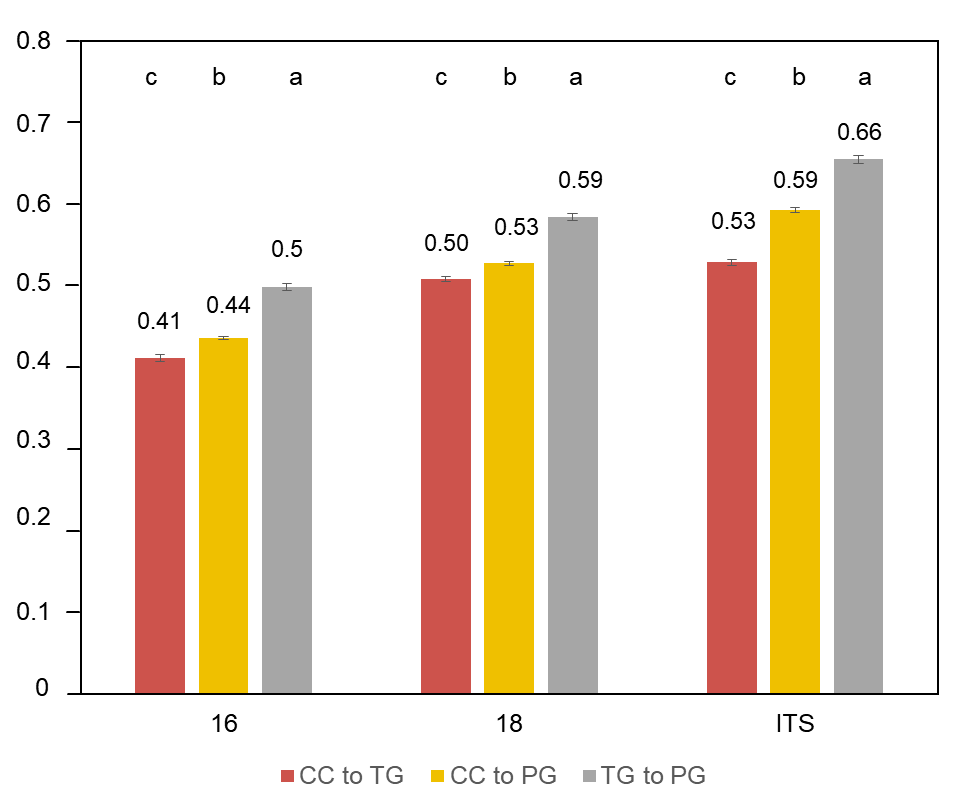
**

**Figure S3.** Bray-Curtis distances between land uses (mean ± s.d.). Different letters above the bars indicate significant differences according to Tukey’s test (p-value < 0.05).

**
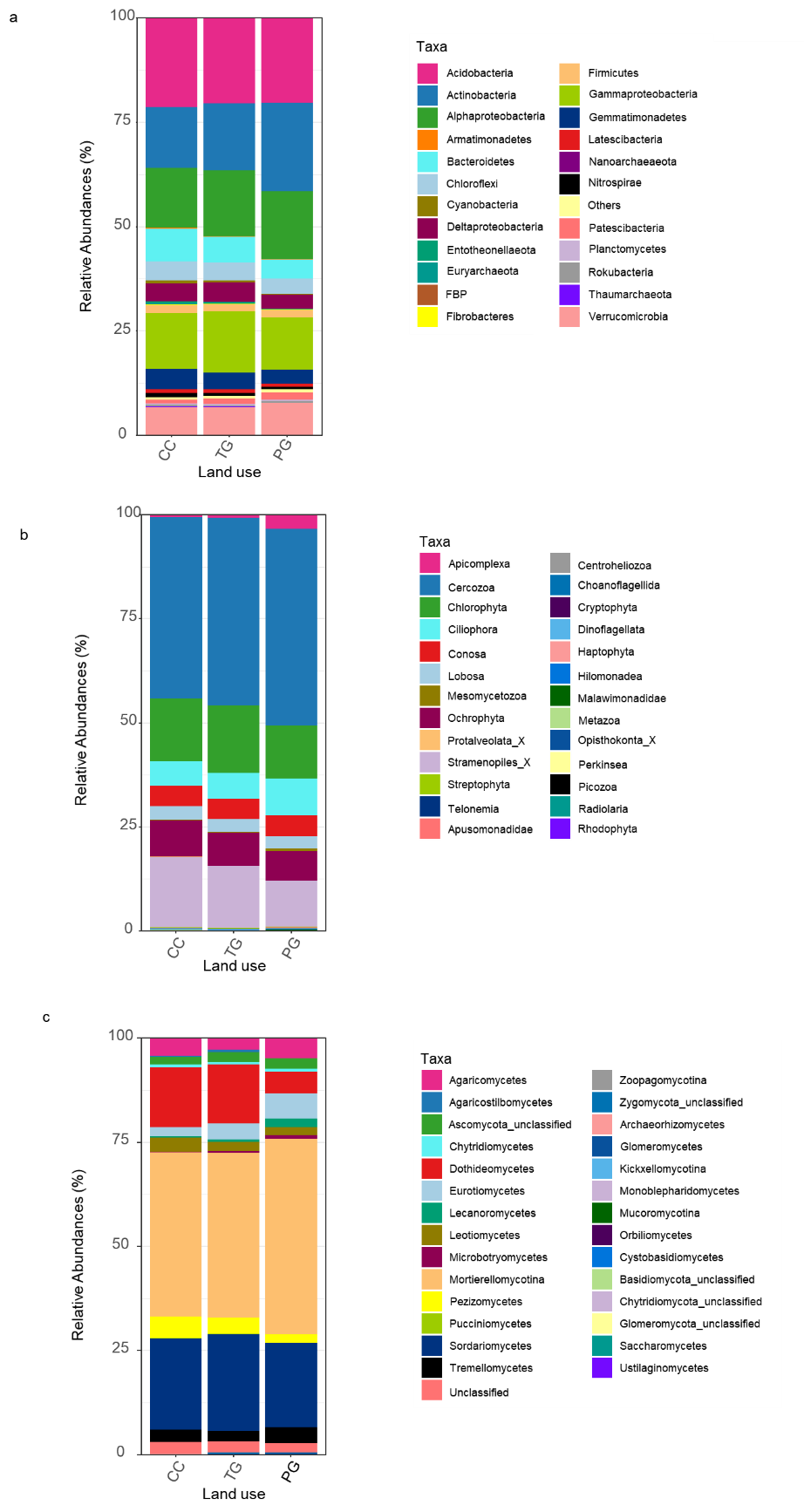
**

**Figure S4.** Microbial composition in continuous cropping (CC), temporary grassland (TG) and perennial grassland (PG) of bacterial (a), protist (b) and fungal (c) communities. Relative abundances are shown at the phylum and class levels and expressed as a percentage of the total number of OTUs.

**
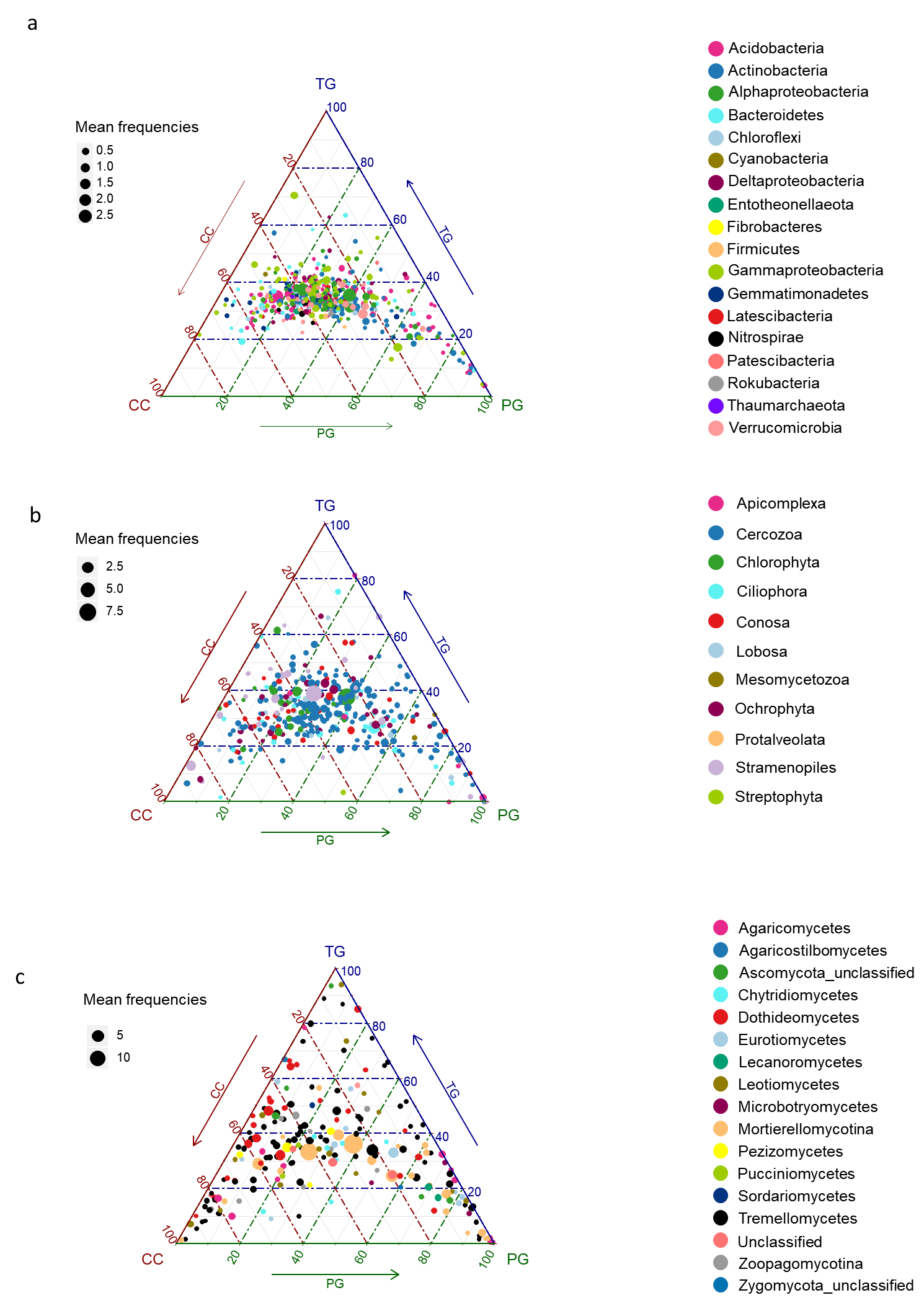
**

**Figure S5.** Ternary plots representing the composition of the microbial community under different land uses. The distributions of the most abundant OTUs in the bacterial (a), protist (b) and fungal (c) communities are shown. The position of each circle on the axis represents the contribution of the indicated land use to the relative abundance of each OTU. The size of the circle indicates the mean frequencies of each OTU in all samples. The colors indicate the affiliation of OTUs at the phylum or class levels.

**
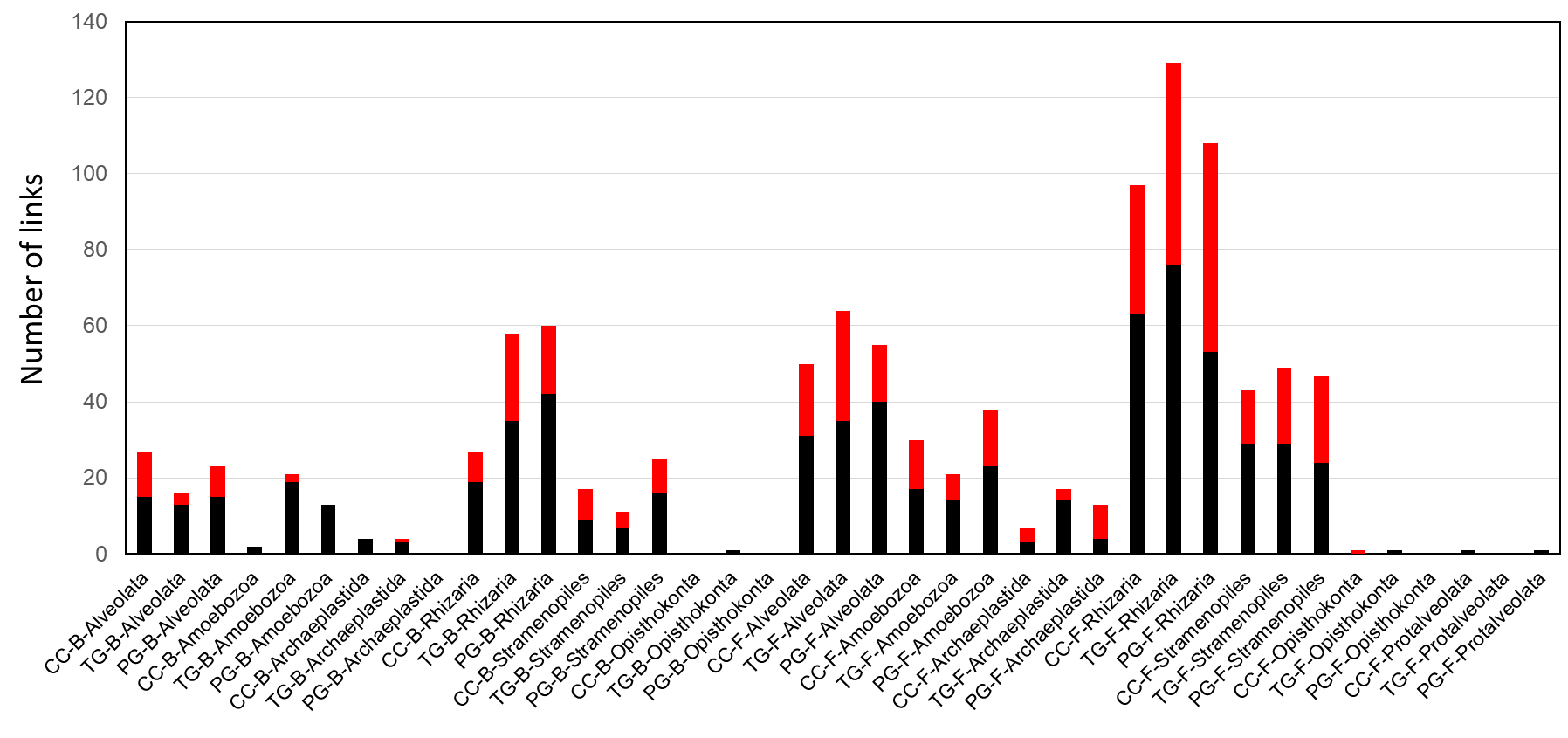
**

**Figure S6.** Number of positive (black) and negative (red) links between bacteria (B) or fungi (F) and groups of protists.
