# Supplementary material for: Land-use intensification differentially affects bacterial, fungal and protist communities and decreases microbiome network complexity
Source: Environ Microbiome. 2022 Jan 6;17:1. doi: 10.1186/s40793-021-00396-9 (PMC8740439; doi:10.1186/s40793-021-00396-9)
Supplement: Supplementary file 2 — Additional file 2: Script 1. R code for differential abundance analysis using DESeq2 package. Script 2. R code of network analysis using a sparse multivariate Poisson log-normal (PLN) model. [file 40793_2021_396_MOESM2_ESM.docx]

**Additional File 2**

***Land-use intensification differentially affects bacterial, fungal and protist communities and decreases microbiome network complexity***

Sana Romdhane^1^, Aymé Spor^1^, Samiran Banerjee^2,3^, Marie-Christine Breuil^1^, David Bru^1^, Abad Chabbi^4,5^, Sara Hallin^6^,  Marcel GA van der Heijden^2,7^, Aurélien Saghai^6^, Laurent Philippot^1*^

^1^University Bourgogne Franche Comte, INRAE, AgroSup Dijon, Department of Agroecology, Dijon, France

^2^Agroscope, Plant-Soil Interactions Group, Zurich, Switzerland

^3^Department of Biological Sciences, North Dakota State University, Fargo 58102, USA

^4^ECOSYS, UMR INRAE, AgroParisTech, Thiverval-Grignon, France

^5^CNRS, Institute of Ecology and Environmental Sciences-Paris (iEES-Paris, UMR Sorbonne Université, CNRS, INRAE), Thiverval-Grignon, France

^6^Swedish University of Agricultural Sciences, Department of Forest Mycology and Plant Pathology, Uppsala, Sweden

^7^University of Zurich, Department of Plant and Microbial Biology, Zurich, Switzerland

^*^Corresponding author: Laurent.philippot@inrae.fr

**Script 1.** R code for differential abundance analysis using DESeq2 package.

library(DESeq2)

#countdata: a table with the read counts

countdata<-read.csv("16S_OTU_table.csv",

row.names=1, sep = ";", check.names=FALSE)

#coldata: a table with metadata on the count table's columns

coldata<-read.csv("myMapping_File.csv",

row.names=1, sep = ";", check.names=FALSE)

dim(coldata)

dim(countdata)

head(coldata)

rownames(coldata) <- coldata$SampleID

colnames(countdata) <- coldata$SampleID

LandUse <-as.factor(coldata[ , "LandUse"])

head(coldata[ , "LandUse"] )

#now construct the data object from the matrix of counts and the metadata table

## The full model was specified previously with the `design = ~ sampletype`:

ddsFullCountTable <- DESeqDataSetFromMatrix(

countData = countdata,

colData = coldata,

design = ~LandUse)

dds <- ddsFullCountTable

as.data.frame( colData(dds) )

dds <- DESeq(dds)

dds

res1 <- results(dds, contrast=c("LandUse", "TG", "PG"))

res2 <-results(dds, contrast=c("LandUse", "CC", "PG"))

res3 <- results(dds, contrast=c("LandUse", "CC", "TG"))

#red fitted curve

#blue: final disversion estimates

plotDispEsts(dds)

plotMA(res2, ylim = c(-1, 1) )

hist(res2$pvalue, breaks=50, col="grey" )

res1 <- res1[ which(res1$padj < 0.00001 ), ]

res1

res2 <- res2[ which(res2$padj < 0.00001 ), ]

res2

res3 <- res3[ which(res3$padj < 0.00001 ), ]

res3

CC_vs_TG <- data.frame(OTU=rownames(res3))

str(CC_vs_TG)

CC_vs_PG <- data.frame(OTU=rownames(res2))

str(CC_vs_PG)

TG_vs_PG <- data.frame(OTU=rownames(res1))

str(TG_vs_PG)

df<- merge(CC_vs_TG, CC_vs_PG, by = "OTU", all = TRUE)

All_DiscriminantOTUs_16S <- merge(df, TG_vs_PG, by = "OTU", all = TRUE)

dim(All_DiscriminantOTUs_16S)

**Script 2.** R code of network analysis using a sparse multivariate Poisson log-normal (PLN) model.

### # Package vignette: https://pln-team.github.io/PLNmodels/articles/PLNnetwork.html

library(PLNmodels)

# import OTU table, mapping file and taxonomy

OTU_table <- read.table("16S_OTU_table.txt", header = TRUE)

info_F <- read.table("myMapping_File.txt", header = TRUE, comment.char = "")

dim(OTU_table)

dim(info_F)

otus_F <- read.table("OTU_taxa.txt", header = TRUE, comment.char = "")

dim(otus_F)

### ## Covariates: take into account the effects of environmental covariates

Block <- as.factor(info_F$Block)

### ## Offset:

abondance=t(as.matrix(OTU_table))

hist(abondance)

Offset <- compute_offset(abondance, offset = "TSS")

Offset

### ## Inferring a network from a count data using Sparse Poisson lognormal model for sparse covariance inference for counts (aka PLNnetwork.)

models_net <- PLNnetwork(abondance~ 1+ Block + offset(log(Offset)))

models_net

### #Extract the best model according to the method [getBestModel()](https://pln-team.github.io/PLNmodels/reference/getBestModel.PLNPCAfamily.html)and StARS, which performs resampling to evaluate the robustness of the network along the path of solutions.

model.StARS <- models_net$getBestModel("StARS")

lambda <- models_net$penalties

#model StARS

model.StARS$plot_network()

#Adjacency matrix with partial correlation

adjacency.StARS <- as.matrix(model.StARS$latent_network())

hist(adjacency.StARS)

hist(adjacency.StARS[adjacency.StARS!=0])

# Extract the edges based on the partial correlation

diag(adjacency.StARS)=0

seuil=0.1 # Partial correlation

adjacency2=adjacency.StARS

adjacency2[lower.tri(adjacency2,diag=T)]=0

edges2 <- which(abs(adjacency2)>seuil,arr.ind=TRUE)

val_edges = adjacency2[edges2]

otus.edges2 <- cbind(rownames(adjacency2)[edges2[,1]],rownames(adjacency2)[edges2[,2]])

head(otus.edges2)

otus.edges2.final=cbind(otus.edges2,val_edges)

write.csv(otus.edges2.final,file = "otu.valedges_forcytoscape.csv")

# Extract the nodes

otus.vertices <- unique(c(rownames(adjacency2)[edges2[,1]],rownames(adjacency2)[edges2[,2]]))

length(otus.vertices)

otus.vertices

write.csv(otus.vertices ,file = " otus.vertices_forCytoscape.csv")

otus_identity <- otus_F[otus.vertices,1]

# Edges and nodes

otus.network=lapply(1:length(otus.vertices), FUN=function(k) otus.edges2[which(otus.edges2[,2]==otus.vertices[k]),1])

names(otus.network)=otus.vertices

otus.network
